# The Size of Endotracheal Tube and Sore Throat after Surgery: A Systematic Review and Meta-Analysis

**DOI:** 10.1371/journal.pone.0074467

**Published:** 2013-10-04

**Authors:** Baoji Hu, Rui Bao, Xiaolin Wang, Shanshan Liu, Tianzhu Tao, Qun Xie, Xiongwei Yu, Jinbao Li, Lulong Bo, Xiaoming Deng

**Affiliations:** Department of Anesthesiology, Changhai Hospital, Second Military Medical University, Shanghai, China; Massachusetts General Hospital, United States of America

## Abstract

**Background:**

Recent studies showed that sore throat following endotracheal intubation was a common problem following surgery. The objective of this systematic review and meta-analysis of published randomized controlled trials (RCTs) or cohort studies was to estimate whether the size of endotracheal tube (ETT) affects the incidence of postoperative sore throat (POST) after general anesthesia.

**Methods:**

The following databases were searched electronically: PubMed (updated to Dec 2012), EMBASE (updated to 15 Dec 2012), Google scholar, World Health Organization International Clinical Trials Registry Platform (Jul 2011), Chinese BioMedical Literature Database (1978 to Jul 2011), and China National Knowledge Infrastructure (1994 to Jul 2011). Studies comparing the size of endotracheal tube for elective surgery were included.

**Results:**

Three trials with a total of 509 female patients were included in the current analysis. The size of ETT used were 6.0 mm and 7.0 mm. Pooled studies from these trials showed that the smaller size of ETT (6.0 mm) significantly decreased the incidence of POST in post-anesthesia care unit (PACU) (RR = 0.56, 95% CI 0.42–0.75, *P*<0.01) and at 24 h after surgery (RR = 0.69, 95% CI 0.48–0.99, *P*<0.05). A smaller size of ETT (6.0 mm) was associated with a lower incidence of PH in PACU (RR = 0.69, 95% CI 0.55–0.87, *P*<0.01), but did not affect the incidence of PH at 24 h after surgery (RR = 0.73, 95% CI 0.46–1.15, *P*>0.05).

**Conclusion:**

Our meta-analysis suggests that patients under general anesthesia with a smaller size of ETT (6.0 mm) were associated with a lower incidence of POST in female patients. More studies with adequate numbers of patients were warranted to evaluate other size of ETT on the incidence of PH and POST after general surgery among different populations.

## Introduction

Anesthesia is considered to be safe and stable, with a very low incidence of mortality and major morbidity [1]. However, minor complications continue to be common and sometimes prolong the recovery time of patients and become a cause of patient dissatisfaction [2]. Endotracheal tube (ETT) is often necessary to achieve airway control during general anesthesia. However, postoperative sore throat (POST) is considered as a common adverse event after general anesthesia with ETTs. POST continues to be reported with a high frequency and can sometimes persist for several days [3]. The incidence of POST ranges from 21% to 71.8% [4]–[6], while the incidence of postoperative hoarseness (PH) is between 40% and 50% [3], [7].

It is known that the size of ETT makes a direct impact on the incidence of POST [3] and PH [8]. Several studies have evaluated the effects of different size of ETT on the incidence of POST. Jaensson M *et al* [3] found that using a smaller-sized ETT can alleviate sore throat and discomfort in women at the post-anesthesia care unit (PACU). Stout *et a*l [9] demonstrated that reducing the size of endotracheal tubes resulted in a significant decrease of the incidence of POST. To our best knowledge, no previous systematic review or meta-analysis were conducted to define the effect of a smaller size of ETT on the incidence of POST and PH. Therefore, we attempted to summarize the available randomized control trials (RCT) and cohort studies to illustrate whether a smaller size of ETT was associated with a lower incidence of POST and PH.

## Materials and Methods

### Data sources and searches

We searched PubMed (updated to Dec 2012), EMBASE (updated to 15, Dec 2012), Google scholar, World Health Organization International Clinical Trials Registry Platform (Jul 2011), Chinese BioMedical Literature Database (1978 to Jul 2011), and the China National Knowledge Infrastructure (1994 to Jul 2011). The Medical Subject Heading and the appropriate corresponding keywords, “endotracheal tube size” AND “postoperative sore throat”. We restricted the findings of the above searched with a highly sensitive search strategy recommended by the Cochrane Collaboration for identifying RCTs [10]. We also checked the reference lists of RCTs and previous meta-analyses identified by the above searches to include other potential eligible trials. Finally, references from relevant articles were reviewed to identify additional studies. Although there was no language restriction, all studies included in this systematic review were published in English. We followed the guidelines of Preferred Reporting Items for Systematic Reviews and Meta-Analysis for reporting our results [11]. There was no protocol exist for our current review.

### Study selection

We identified and reviewed all studies that met the following criteria: RCT or cohort study; population: adults undergoing general anesthesia; intervention: more than one different size of ETT; and outcome: incidence of POST or PH.

### Data extraction and assessment of study quality

The selection of studies for inclusion in the review was performed independently by the reviewers (Hu and Bao) after using the search strategy described previously. Data were abstracted independently by Hu and Bao by using a standardized data collection form. There was no attempt to bind to the reviewers (Hu and Bao) to the authors or the results of the relevant trials. Details of study designs (i.e., date, location and sample size), patient characteristics (i.e., population gender), study design (i.e., inclusion/exclusion criteria, ETT insertion and anesthetic technique), intervention (i.e., definition of ETT size), surgery duration, anesthesia maintenance narcotic and main outcomes were collected. If data needed clarification or was not present in the publication, the original authors were contacted. Extracted data were entered into Microsoft Office Excel 2007 and were checked by the third author (Wang). Discrepancies were resolved by discussion, or advice was sought from a third author.

The primary outcome of the data was the incidence of POST in PACU and at 24 h after surgery. The secondary outcomes were the incidence of PH in PACU and at 24 h.

### Statistical analysis

Analyses were on an experiment-to-control basis. Differences were expressed as relative ratios (RRs) with 95% confidence intervals (CIs) for dichotomous outcomes. A fixed-effect model was used and a random-effects model was employed in the case of significant heterogeneity (*P*-value of chi-square test less than 0.10 and *I* ^2^ greater than 50%). Potential sources of heterogeneity were identified by sensitivity analyses conducted by omitting one study in each turn and investigating the influence of a single study on the overall pooled estimate. Publication bias was assessed by visually inspecting funnel plots. A *P* value of less than 0.05 was considered statistically significant. All statistical analyses were performed using Review Manager, version 5.0 (RevMan, The Cochrane Collaboration, Oxford, United Kingdom).

## Results

### Identification and selection of study

**Figure 1:**
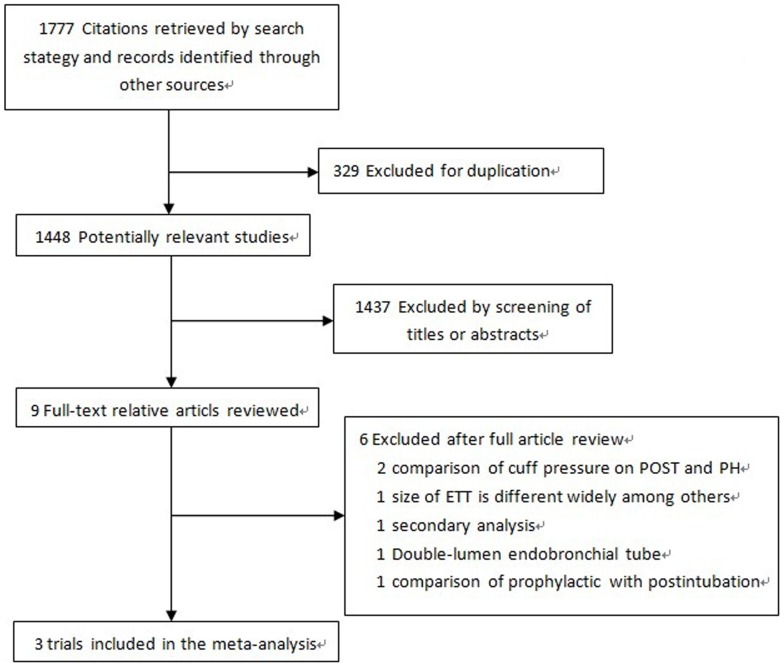
Flow chart detailing retrieved, excluded, assessed, and included trials.

The comprehensive search yielded a total of 1,976 relevant publications, and the abstracts were obtained for all citations (Figure 1).Nine RCTs with a total of 1,753 patients were identified [3], [12]–[19], while six of those were extracted for reasons demonstrated in Figure 1. Finally, three trials [3], [12], [19] with a total of 509 patients were included for further analysis. The Cohen ??? statistic for agreement on study inclusion was 0.92.

### Study characteristics and quality

Among the three trials, one was conducted in Asia [19], two in Europe [3], [12]. All trials were published in English. The mean age of patients ranged from 45 to 59 years. The selected trials examined various surgery types, including plastic surgery [3], [12], ear nose and throat (ENT) surgery [3], [12], general surgery [12], [19], orthopaedic surgery [12], urology [12], eye surgery [12], gynecological surgery [12]. The gender of all patients included in this analysis was female, and the definition of a smaller and larger ETT size was 6.0 mm and 7.0 mm in diameter, respectively. No double lumen ETT was used. There was no movement of ETT during surgery. The cuff of the ETT was filled with air and the cuff pressure was under 30 cmH_2_O among all included studies. The naso-gastric tube was not applied in all studies. Characteristics of the included trials and summary of the size of ETT on POST and PH were presented in Table 1.

**Table 1:** Characteristics of the included trials and summary of the size of ETT on POST and PH.

Study	Gender	Mean age(year)	Type of Surgery	Cuff pressure (cmH_2_O)	Other measures of ETT	Movement of ETT	Anesthesia duration (min)	Size of ETT	Sample size	POST in PACU	POST at 24 h	PH in PACU	PH at 24 h
**Jaensson 2010**	Female	45–50	Plastic, ENT	20–30	No	No	>190	6.0	48	10	9	18	14
								7.0	49	24	10	23	20
**Jaensson M 2012**	Female	>17	General, Orthopaedic, Urology, Eye, Gynaecological, Plastic, ENT, Hand	21–30	No	No	>135	6.0	60	14	22	7	9
								7.0	60	25	35	15	12
**Xu 2012**	Female	33–59	Thyroid	<25	No	No	39–89	6.0	263	24	/	148	/
								7.0	29	15	/	23	/

ETT = endotracheal tube, POST = postoperative sore throat; PACU = post-anesthesia care unit; PH = postoperative hoarseness; ENT = ear, nose and throat; / = none reported.

**Figure 2:**
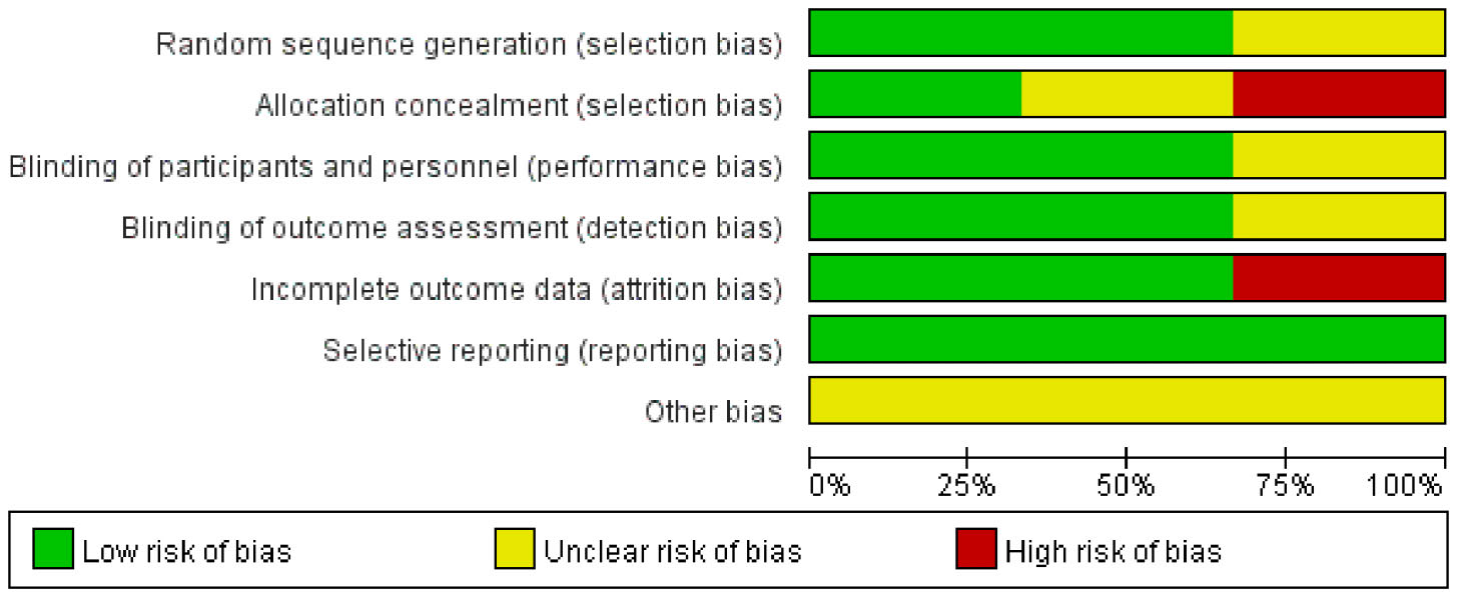
Risk of bias graph: review authors' judgments about each risk of bias item presented as percentages across all included studies.

**Figure 3:**
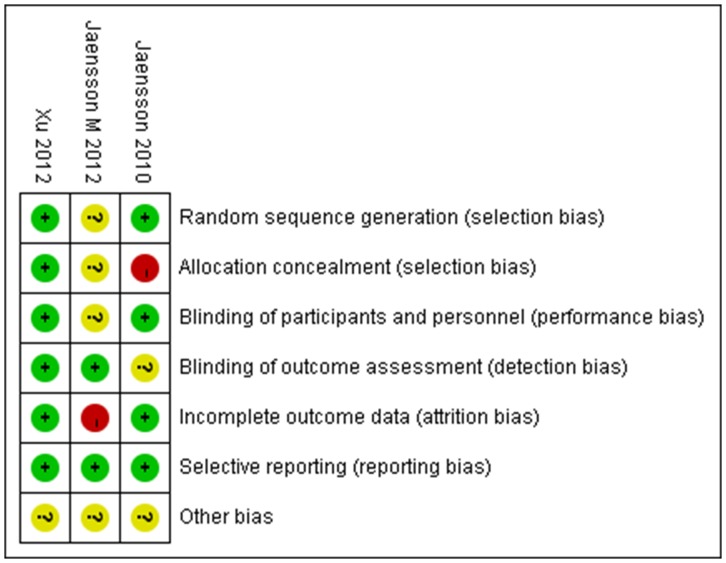
Risk of bias summary: review authors' judgements about each risk of bias item for each included study.

Among all selected trials, one is a cohort study [12]. Randomized sequence and allocation sequence concealment were adequately conducted in two studies [12], [19]. Blinded fashion was clearly stated in the adjudication of POST in two studies [12], [19]. The numbers and reasons for withdrawal or dropout were reported in details in all trials. An overview of the risk of bias was shown in Figure 2 and Figure 3.

### Primary outcomes

**Figure 4:**
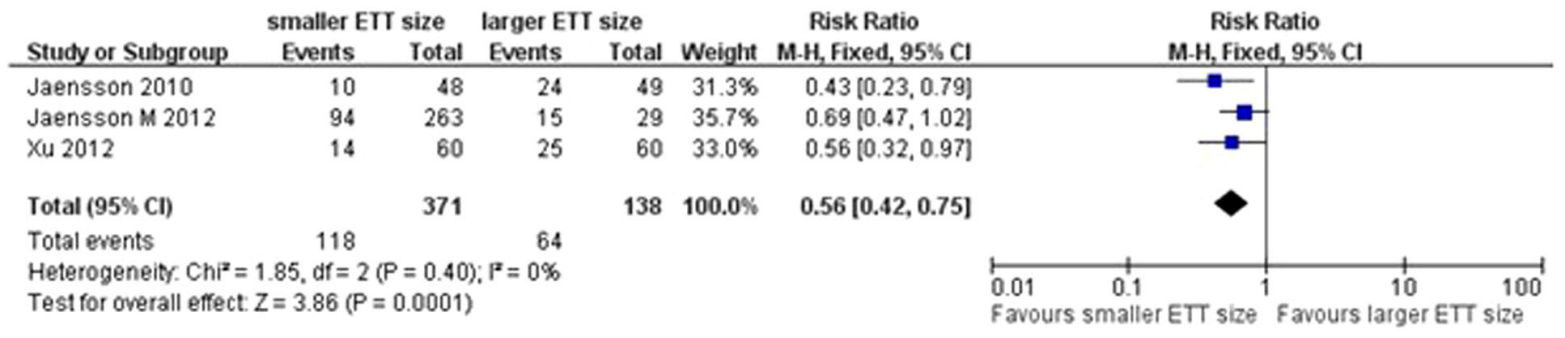
Forest plot of comparison of POST in PACU.

**Figure 5:**
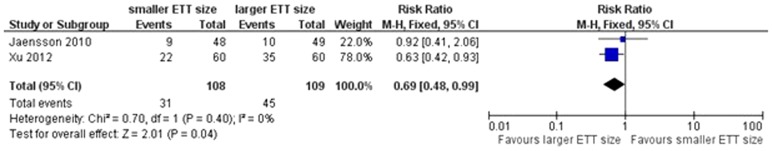
Forest plot of POST at 24

Data on primary outcomes were available in three trials [3], [12], [19] (n = 509). Our meta-analysis indicated that a smaller size of ETT could significantly reduce the incidence of POST in PACU(RR = 0.56, 95% CI 0.42–0.75, *P* = 0.0001; *P* for heterogeneity = 0.40, *I* ^2^ = 0%; Figure 4) and at 24 h after surgery (RR = 0.69, 95% CI 0.48–0.99, *P* = 0.04; *P* for heterogeneity = 0.40, *I* ^2^ = 0%; Figure 5).

### Secondary outcomes

**Figure 6:**
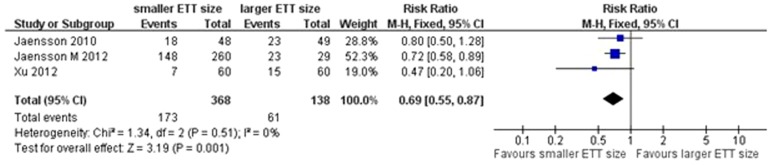
Forest plot of comparison of PH in PACU.

**Figure 7:**
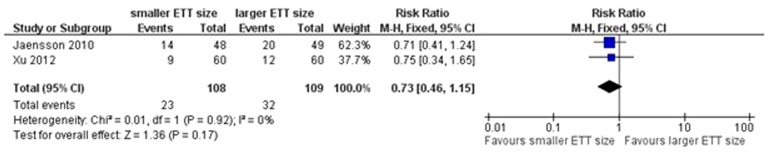
Forest plot of comparison of PH at 24

Our analysis indicated that a smaller size of ETT was associated with a lower incidence of PH in PACU (RR = 0.69, 95% CI 0.55–0.87, *P* = 0.001; *P* for heterogeneity = 0.51, *I* ^2^ = 0%; Figure 6). The incidence of PH at 24 h after surgery was reported in two trials [3], [19]. Our pooled analysis indicated that the size of ETT did not affect the incidence of PH at 24 h after surgery (RR = 0.73, 95% CI 0.46–1.15, *P* = 0.17; *P* for heterogeneity = 0.92, *I* ^2^ = 0%; Figure 7).

### Sensitivity analysis and publication bias

**Figure 8:**
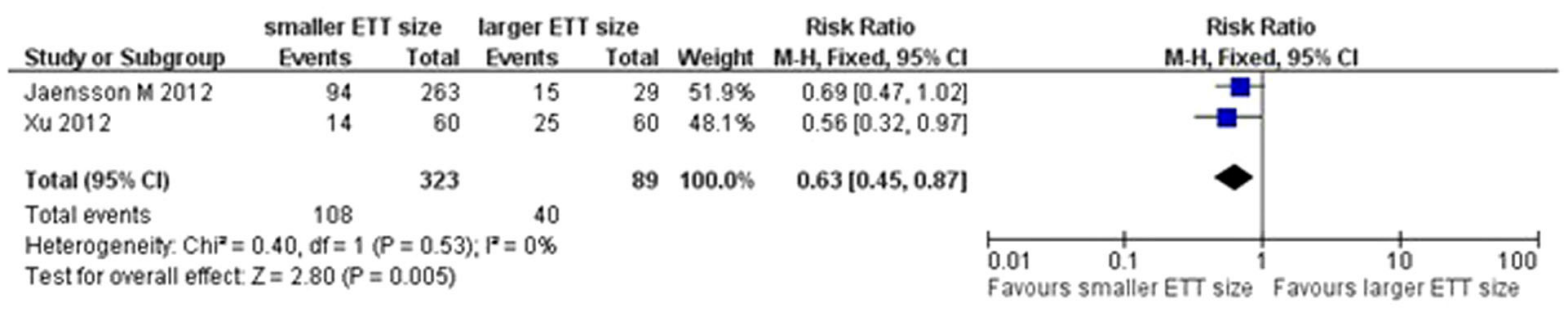
Forest plot of comparison of sensitivity analysis.

**Figure 9:**
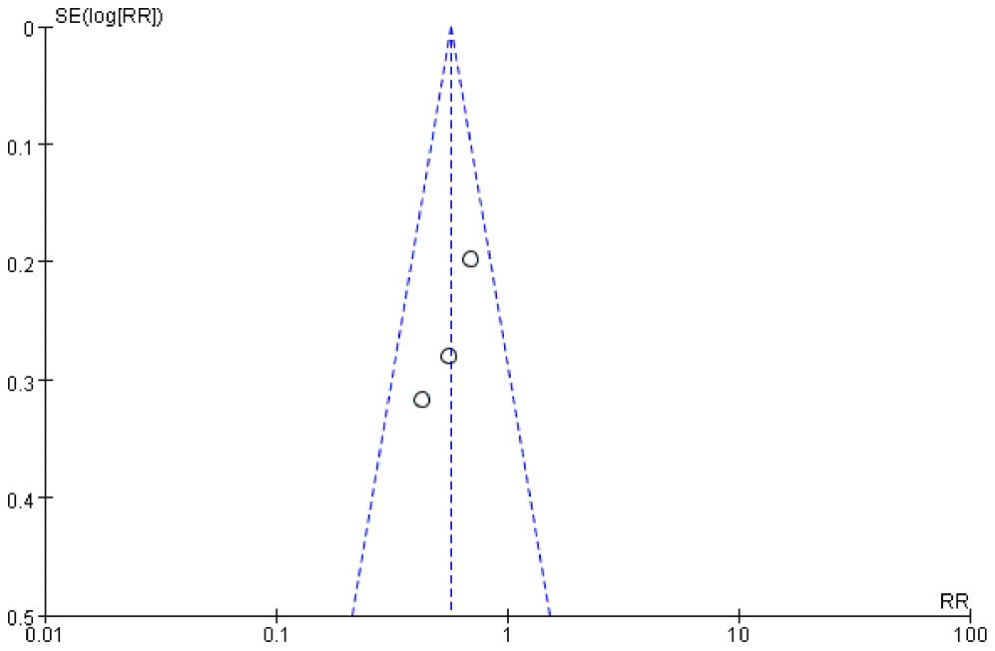
Funnel plot of trials of POST in PACU.

**Figure 10:**
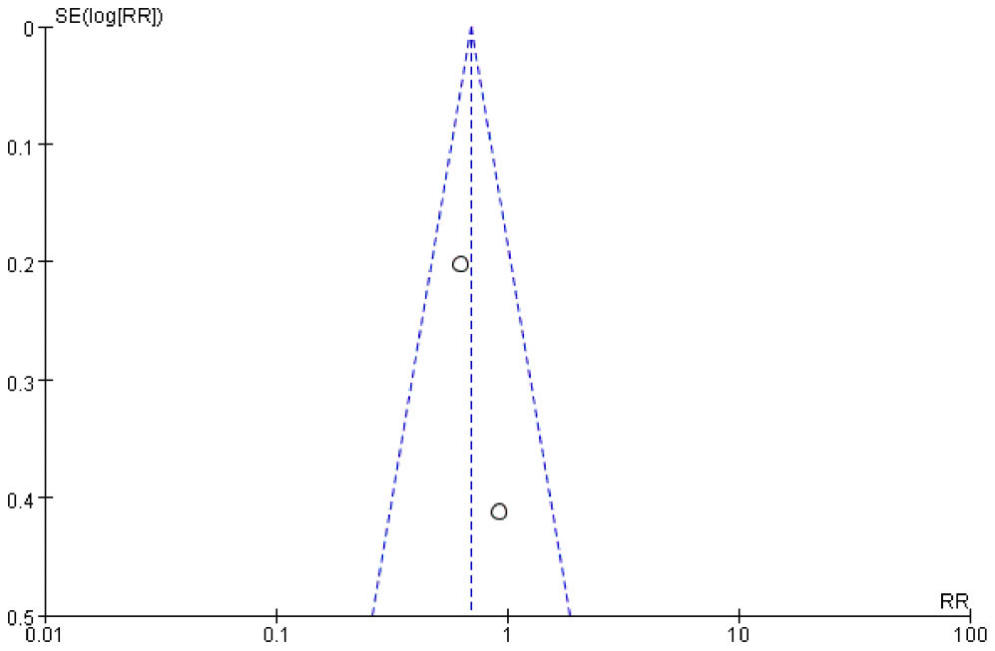
Funnel plot of trials of POST at 24

Tests for heterogeneity identified the trial by Jaensson (2010) *et al* with outlying results. Exclusion of this trial resolved the heterogeneity, but did not change the results (POST in PACU: RR = 0.63, 95% CI 0.45–0.87, *P* = 0.005; Figure 8). For the result of POST in PACU and at 24 h after surgery, there was no evidence of significant publication bias by inspection of the funnel plot (Figure 9 and Figure 10).

## Discussion

To our best knowledge, this is the first meta-analysis of studies evaluating the different size ETT for preventing POST. Our meta-analysis suggests that a smaller size of ETT (6.0 mm) could significantly reduce the incidence of POST in PACU and at 24 h after surgery. In addition, the present study shows that a smaller size of ETT (6.0 mm) reduced the incidence of PH in PACU, but not at 24 h after surgery.

POST usually occurs in intubated patients and is one of the most common complaints from patients after endotracheal extubation [6]. The underlying causes of POST include mechanical pressure by the cuff of the tube. It's known that larger size of ETTs exert higher pressure at the tube mucosal interface and might lead to a greater area of mucisal trauma [20]. Stout *et al* [9] showed that the incidence of POST and PH was reduced by the use of smaller tubes (6.5 mm for women) compared with larger ones (8.5 mm for women). We did not include this study due to potential bias that patients with a 8.5 mm tube might be with a higher incidence of POST than patients with a 7.0 mm tube. In addition, the movement of cuff and tube in the trachea from the location when positioning and manipulation of goiter during surgery is responsible for POST. It is recommended that the cuff pressure of ETT should between 15 to 25 cmH_2_O [21].

Several methods have been proposed to reduce the incidence of POST. One previous study conducted by Porter *et al* [16] showed that the use of lidocaine to inflate the endotracheal tube cuff didn't appear to be effective in decreasing the incidence or severity of POST compared with the use of saline or air. However, a recent systematic review, including 1232 patients from 15 studies, indicating that topical and systemic lidocaine could reduce the risk of POST [22].

Female gender is a risk factor for POST. Jaensson *et al*'s [12] study indicated that there is a significant increase of the incidence of POST in women (n = 292, *P* = 0.028), while this was not significant in men. Another factor which was thought to be associated with an increased risk for POST was the experience of the anesthesia personnel [12]. However, this factor has not been shown to increase the risk for POST in previous studies [6], [23], [24]. It is likely that training on a manikin during the early phase of employment may improve the technique and thereby reduce the risk of POST.

There were several limitations in the present study. First, the geographic regions covered Europe (Sweden), and Asia (China). Therefore, our results limited generalizability to the regions. Second, there was considerable heterogeneity among the included trials. The target population varied greatly. The adopted definitions of POST differed from 1 h to 24 h or even to 96 h. Furthermore, one study conducted by Jaensson *et al* [12] was a prospective observational cohort study, but not an RCT. However, the study was conducted in a blinded manner and applied the allocation sequence concealment, which might decrease potential bias of the analysis. Due to limited data available, we determined to include this study.

Due to considerable heterogeneity as well as a limited number of RCTs regarding the outcomes, caution should be given when interpreting the results. Patients from all included studies were female. Whether the results of our current meta-analysis were applicable to male patients was unknown. Finally, the sizes of ETT used in all included studies were 6.0 mm and 7.0 mm. The effect of other size of ETT on the incidence and severity of PH and POST needs to be clarified in future.

## Conclusions

Our meta-analysis suggested that a smaller size of ETT could reduce the incidence of POST in PACU and at 24 h after surgery in female patients. In addition, a smaller size of ETT was associated with a lower incidence of PH in PACU, but not at 24 h after surgery. Further studies with rigorous design and adequate samples were needed to clarify the effect of different size of ETT on the occurrence of POST and PH among different populations in future.

## Supporting Information

Checklist S1 **PRISMA Checklist.**
(DOC)
